# Remarks on Hydrencephalus

**Published:** 1816-02

**Authors:** G. D. Yeats


### *For the London Medical and Physical Journal.*

### *Remarks on Hydrencephalus;* by G. D. Yeats, M.D.

THE Editors of the Eclectic Review* having, with much gentlemanly candour and critical acumen, noticed my pamphlet on Water in the Brain, and having stated what they conceive to be my attachment to some particular doctrine, I am desirous of making a few remarks, in consequence of the distinctive divisions they have drawn between these doctrines, and likewise on account of my supposed too near approach to the *verba magistri.* The objections, too, which have been urged from so very respectable a quarter, and with such evident zeal in the cause of science, cannot pass unheeded; although they are, in some of the observations, so qualified as to approach near to the opinions I have advanced, particularly on the subject of hydrencephalus.

The doctrines which have, at different times, prevailed in the schools of physic, have, for the most part, taken their origin from the labours of some industrious individual, who, by the force of native talents, aided by the facilities of education and persevering investigation, by the improvements of science, and the partial discoveries of his predecessors, acquires a tone of authority to his opinions, and of sanction to his system. The preceding doctrines are thus gradually disregarded, and become ultimately neglected, from the clearer light in which the subject is viewed by the recent investigations and improvements, and from the additional insight which is given into some of the recesses of science, hitherto not sufficiently explored; and the new system is erected upon what is considered at the time a firm foundation. Thus it was that the doctrines of the Galenic school disappeared before the flood of light which the discoveries of Hervey poured upon physiology. The doctrines of Boerhaave, derived from the humeral pathology, were, in like manner, made to yield to the more philosophical and consistent reasoning of Hoffman, founded upon the more solid basis of anatomical enquiry; and it is not a little re-

---

* Eclectic Review, published Sept. 1, 1815.

markable, markable, that the doctrines of the Boerhaavian school, in our celebrated northern University, were overturned, about seventy years ago, by the medical students, who had procured the works of Hoffman. The professors at that time had all been the pupils of Boerhaave; but the sanction of age and authority could not shield the humoral pathology from the attacks which were brought against it, by those who were armed with improvements from an increased knowledge of anatomy. The opinions which have since prevailed have been only modified from those of Hoffman, whose works are a rich and inexhaustible mine. The labours of Brown, though evidently the efforts of genius and intellectual endowment, proved abortive, by resolving every thing into debility or tone. Thus the Brunonian disciple had nothing to perform but the simple task of adapting his medicines to the one or the other state, forgetting there is an accompanying movement in the constitution, which imperiously calls for weighty consideration, before you can, with ultimate success, subtract from energetic action, or add to the tone of the system. The unwarrantable extent to which system-makers, or their followers, have carried the tenets upon which they have founded their doctrines, however cautious it ought to make us in receiving implicitly the principles of any justly distinguished physiologist, should not, however, induce us altogether to reject the foundation upon which that system may have been built. Something may be culled from all of practical advantage, if judiciously selected and discriminately applied. In this way the verba magistri, carefully separated from the works of our experienced predecessors, and properly employed to confirm the judicious observations of our contemporaries, founded upon experimental inquiry, or to refute the eccentric theories to which that experimental inquiry may have given rise, become of essential use. Thus an accumulation of valuable information from others is added to our own stock of knowledge, and much precious time is saved by not investigating that which has already been abundantly confirmed. "I never read any thing," said a celebrated anatomist, now dead, "except the book of Nature, for it is only there you can acquire useful knowledge:"—"then," answered the gentleman, with considerable acuteness, to whom this observation was made, "then you should not write, sir; for, upon the same grounds, nobody should read your writings."

In consequence of some particular circumstances which occurred in the early part of my professional career, I was induced to turn my thoughts more closely to the consideration of water in the brain. I was not satisfied with the explanation of the symptoms I read in books, because it did not appear to me to accord with the phenomena; and I was still less satisfied with the mode of treatment, for the melancholy reason that it was almost uniformly unsuccessful. I then began more accurately to analyse the symptoms with diffident and anxious eagerness, and to be more minute in my inquiries respecting the commencement of the complaint; and it was not long before I perceived that, in by far the majority of cases, symptoms which I then considered singular and contradictory, preceded the attack on the brain. Whilst my mind was agitated with thoughts on this subject, the works of Rush, and, subsequently, those of Hamilton, of Abernethy, and of Cheyne, fell in my way: the perusal of these, with a reference to the labours of Hoffman, of Cheston, and of Kirkland, contributed to confirm my suspicion that hydrencephalus was not always originally a disease of the head, and I accordingly hailed with gladness aid so powerful in assisting me to become emancipated from the received doctrines on the subject. I do not feel attached to systems in physic; they are good to teach the student how to tread in the paths of science, but they should not be trammels to check the ardour of research, or to fetter the excursive flights of the mind. It was thus that the genius of a Hervey and a Sydenham burst asunder the bonds of restrictive law which scholastic discipline had imposed upon medical inquiry; and the mighty mind of a Newton and a Locke swelled beyond the measure of its chains, and took its daring flight into the regions of intellectual immensity.

In publishing my letter to Dr. Wall, I wished to state nothing but those facts which had come within my own knowledge in a practical point of view, and to avail myself of such observations as their publication might call forth from the profession, either to correct or confirm a subject where "shadows, clouds, and darkness rest upon it." The remarks which have been elicited by the publication of this letter, and the approbation which has been bestowed in the criticisms of the Medical Repository, the Edinburgh Medical and Surgical Journal, and also, I may add, by the ingenious writer in the Eclectic Review, have confirmed to me a feeling I entertained that I did not see the facts I have advanced through the prejudiced medium of a biassed mind.*

But, whatever importance I may attach, and it is very considerable, to the idea that hydrencephalus in its origin commences in organs distant from the brain, the Eclectic

* Medical Repository for April 1815; Edinburgh Medical and Surgical Journal for October 1815; and Eclectic Review for September 1815.

Reviewer

Reviewer will see, by turning to page 8 of my Letter, that I am very far from denying that hydrencephalus does originate, how often I cannot say, within the cranium. But experience of every sort, personal, colloquial, and from reading, has amply confirmed to me that, unless we treat the disease as connected with, or arising from, gastro-hepatic derangement, we shall be disappointed in our practical expectations. There is no doubt, however, that the distinction of the derangement in the digestive organs is often only the removal of effects produced by morbid action in the brain: still, however, the proper management of the gastro-hepatic derangement is one great step towards a cure, inasmuch as it obviates or mitigates the reaction upon the brain, and thus prevents an increased intensity and complication of disease.

I very much doubt whether the gradual paralytic state, which slowly creeps upon the constitution in some children, first manifesting itself in a hesitating and imperfect movement of the lower limbs in walking, and causing the child to trip at every the slightest eminence in its way, is to be considered as connected with genuine hydrencephalus, although no disease shall be discovered along the spine from examination. There appears to be, in such cases, some injury in the brain, with great suffering and derangement in the digestive functions.

I have witnessed this disease at various periods of life, and within the last year three or four cases of the kind in adults, at different ages, have come within my knowledge, in which the chylopoietic derangement was very great; and none of them were benefitted without such medicines as directly operate upon the gastro-hepatic functions. One is now at Bath, the waters of which, taken internally, have more than once afforded relief. The eldest of them died in an apoplectic fit, after having become completely jaundiced; and he was in a rank of life in which controul is not so easily attainable. Another has been so far recovered from his palsy and an hysterical *decipiency* of mind as to be able to resume his situation as Clerk in the Bank, from which he had been obliged to absent himself for above three years; and this amendment has been brought about by such remedies as would more particularly affect the digestive organs. He was at one time completely jaundiced, with much tenderness over the region of the liver.*

* This is the case alluded to in p. 99 of my letter on Water in the Brain. The amendment in the palsy has taken place since the publication of the letter, at which time the case was under management.

Within the last two months, a child, four years of age, the interesting daughter of one of our gallant naval defenders, has been brought to town, in whom, as in the cases just alluded to, a paralytic affection has gradually come on, connected with great derangement of the nervous system, and of the digestive organs. Strabismus; great dilatation of the pupils; head-ach; repeated vomitings; frequent recurrences of epigastric fulness, with unhealthy fæcal discharges; much costiveness; inability of guiding the limbs properly when attempting to walk, and that not without assistance; inability also of standing without support; loss of appetite; and an unsteady, intermitting, quick, and weak pulse; formed the assemblage of symptoms in this case. Although a favourable termination may be considered as very doubtful, yet it affords much satisfaction to know that all the symptoms, save the paralysis, which has, however, been mitigated, have been for some time removed by the gastro-hepatic treatment. Powerful tonics had been formerly repeatedly given without any advantage.

I have mentioned these kinds of palsies, on account of the allusion which the candid writer in the Eclectic Review has made to them, as some of the early symptoms which mark the commencement of hydrencephalus. They appear to me not to be connected with that particular morbid action constituting the genuine disease, and sometimes producing effusion into the ventricles of the brain: they mark, however, the good effects to be derived from a proper attention to the healthy action of the digestive organs. The connection, too, between the diseases of the brain and a derangement of the functions of the abdominal viscera, is as conspicuous in the advanced periods of life as in our infant years. In the latter we have hydrencephalus, either rapidly hurrying the little sufferer into a premature grave, or approaching in a lingering form, with a gradually enlarging head: in the former, apoplexy of the sanguineous kind either instantly surprises the patient at the moment of apparent high health and conviviality, or slowly advances upon him in the serous kind with lethargy and carus. The experienced physician knows how much, in both instances of infancy and age, the disease of the brain is connected with an imperfect action in the liver, or in the more immediate digestive organs, or in the intestinal canal. The analogy is striking; the conclusion inevitable, whether we explain it from mechanical or sympathetic causes. The constipated state of the bowels at both periods of life is attended, though not always, in either case, with a morbid affection of the brain. The bulimious appetite which so often exists previous to an attack of genuine nuine apoplexy, and the continuance of it, in the paralytic state, when the patient survives the stroke, is not a little remarkable, when we recollect the kind of appetite that very frequently exists in hydrencephalus: it is an irregular excitement of the stomach—a craving—something more than appetite, which produces an eagerness to eat without a corresponding gratification from its indulgence; and this indulgence is followed by consequences neither creditable to the physician who permits, nor advantageous to the patient who insists upon it.

I might pursue the analogy still further, did not the limits of a paper in a periodical publication check my progress.

The pathologist who refers the origin of diseases constantly to one particular part, must either have a very limited knowledge of anatomy, or must take a very partial view of the animal economy. Whoever, therefore, believes that we are always to look to the liver for the causes of disorders, or that the stomach is uniformly the *primum mobile* in morbid actions, or that irregular intestinal movements is the nucleus upon which are found the various diseases to which we are subject, will most probably find himself disappointed, in only relieving the symptoms, and in not curing the diseases. Whatever division, therefore, may be made of doctrines, as a general principle, into purgative and hepatic theory, or into theory of the digestive organs, they will form a good distinction in systematic writings, but will not be of much use when the prescriber takes the pen in hand, except he keeps in view the reciprocal effects which a morbid action of one portion of the digestive organs has upon another, and ultimately in this way upon the system at large.

*King-street, St. James's-square;*
*Jan.* 1, 1816.

---

*For the London Medical and Physical Journal.*

